# MicroRNAs, TGF-β signaling, and the inflammatory microenvironment in cancer

**DOI:** 10.1007/s13277-015-4374-2

**Published:** 2015-11-12

**Authors:** Lingling Guo, Yongsheng Zhang, Lifeng Zhang, Fengbo Huang, Jinfan Li, Shouli Wang

**Affiliations:** 1 Department of Pathology, School of Biology and Basic Medical Sciences, Soochow University, Suzhou, 215123 China; 2 Department of Pathology, The Second Affiliated Hospital of Soochow University, Suzhou, 215004 China; 3 Department of Surgery, The First Affiliated Hospital of Soochow University, Suzhou, 215006 China; 4 Department of Pathology, The Second Affiliated Hospital of Zhejiang University, Hangzhou, 310009 China; 5 Institute of Radiology and Oncology, Soochow University, Suzhou, 215006 China

**Keywords:** MicroRNA, TGF-β signaling, Inflammatory microenvironment, Cancer

## Abstract

Inflammatory cells and mediators form a major part of the tumor microenvironment and play important roles in the regulation of cancer initiation, tumor cell proliferation, and metastasis. MicroRNAs (miRNAs) play important roles in several physiological and pathological processes, including the regulation of the inflammatory microenvironment in cancer. Transforming growth factor-β (TGF-β) is an inflammation-related cytokine that functions in both tumor suppression and promotion; however, its underlying molecular mechanisms remain unclear. Recent evidence indicates an association between miRNAs and TGF-β signaling, providing new insight into the nature of the inflammatory microenvironment in cancer. The present review is an overview of the interaction between miRNAs and inflammatory cytokines, with emphasis on the cross talk between TGF-β signaling and miRNAs and their influence on cancer cell behavior. The emerging roles of miRNAs in cancer-related inflammation and the potential to target miRNA signaling pathways for cancer therapy are also discussed.

## Introduction of miRNA and the tumor inflammatory microenvironment

MicroRNAs (miRNAs) are endogenous single-stranded non-coding RNAs of 18–25 nucleotides [1] that function in the post-transcriptional regulation of gene expression through translational repression or cleavage of messenger RNA (mRNA) targets in a specific base pairing manner [2]. One miRNA has the potential to affect the expression of several proteins, and one protein can be regulated by several miRNAs. The modulation of oncogenic and anti-oncogenic miRNAs could, in principle, affect the progression of cancer [3–5]. miRNA expression profiling of human tumors has identified signatures associated with diagnosis, staging, progression, prognosis, and response to treatment [6]. Emerging evidence suggests a direct link between miRNAs and cancer [7], inflammation [8, 9], and autoimmune diseases [10–12]. miRNAs regulate immune responses by modulating the expression of immune-related genes.

**Fig. 1:**
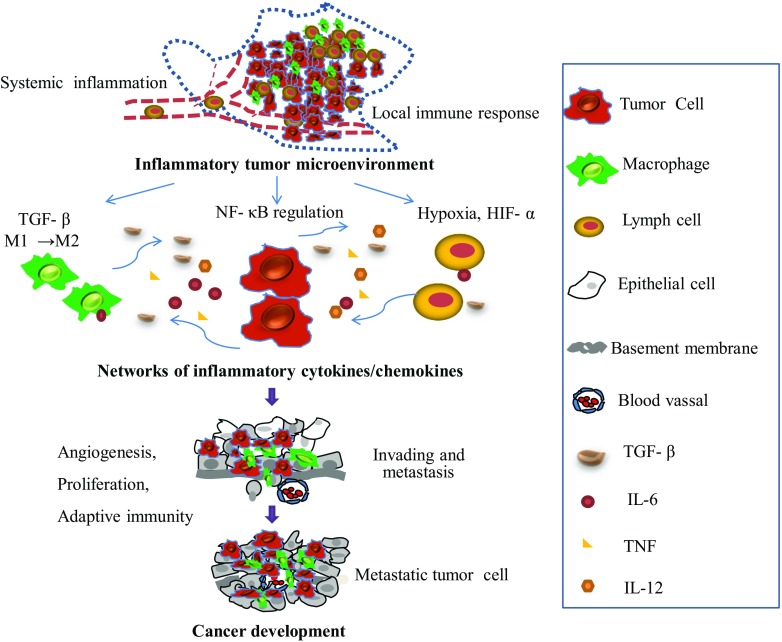
Overview of the interaction of cytokines in the inflammatory tumor microenvironment. Tumor-associated inflammation develops simultaneously with tumor development. The tumor microenvironment is a complex scaffold of various cells and extracellular matrix. Different cells contribute by producing cytokines and respond to stimuli secreted by other cells. This creates a favorable microenvironment for tumor growth and progression. The inflammatory response plays a central role in angiogenesis, tumor proliferation, local immunosuppression, and metastasis

The connection between cancer and inflammation was first identified in the nineteenth century and is currently recognized as one of the six hallmark features of cancer development and progression [13, 14]. The field of cancer-related inflammation is driven by the hypothesis that extrinsic pathways associated with conditions that cause or promote cancer and intrinsic inflammatory pathways activated by genetic events lead to the production of inflammatory cytokines [13, 15]. Inflammatory cells and inflammatory mediators form a major part of the tumor microenvironment, which has been highlighted as an important factor in cancer progression (Fig. 1). For example, CD4(+)CD25(high)Foxp3(+) regulatory T cells (Tregs) enriched in the tumor-associated microenvironment play an important role in cancer immune evasion [16] and are considered potential therapeutic targets in human diseases [17]. However, the complex nature of the inflammatory microenvironment remained unclear until recently, when several miRNAs, such as miR-126, miR-132, miR-146, miR-155, and miR-221, emerged as important transcriptional regulators of inflammatory mediators [8]. Research in the miRNA field opened new horizons in our understanding of the role of the inflammatory microenvironment in cancer. In the present review, we focus on the interaction between miRNAs and inflammatory cytokines in the tumor microenvironment, with particular emphasis on the transforming growth factor-β (TGF-β) signaling pathway. In addition, we present a novel viewpoint based on the modulation of the tumor microenvironment as miRNA-based cancer therapy.

## Control of miRNA biogenesis by inflammatory cytokines

Originally, research aimed at investigating miRNA expression profiles revealed differences in the expression of specific miRNAs in cancers. Recently, research has started to focus on the regulation and function of miRNAs. Several studies have investigated the specific influence of key inflammatory cytokines on miRNA expression. Overexpression of inflammatory cytokines such as tumor necrosis factor (TNF)-α, interleukin (IL)-6, IL-1, IL-8, IL-10, IL-12, and TGF-β can either promote or inhibit cancer development [18, 19]. Several miRNAs such as miR-155 and miR-21 have been implicated in cancer development and inflammation [20], and they are controlled by inflammatory mediators, the most prominent being Toll-like receptors (TLRs), TNF, TGF-β, and other cytokines that link the functions of miRNAs with inflammatory events [11, 20–24]. Table 1 describes the miRNAs implicated in both cancer and inflammation and their functions.

**Table 1:** Representative microRNAs implicated in both cancer and inflammation

miRNA	Cancer/inflammation	Function	Related cytokines and ranscriptional factors	Ref.
miR-155	Leukemias/lymphomas, breast, colon, lung, pancreatic, and gastric tumors	Promote the progression of inflammatory pathologies; targeting of key oncogenic suppressors or anti-inflammatory signal transduction pathways	TNF-a; IFN-γ/IL-12/IL-18	[25–29]
miR-125b	Colorectal cancer (CRC), muscle-invasive bladder cancer (MIBC), ovarian cancer	A promising diagnostic biomarker for CRC and a promising prognostic parameter for MIBC; targeting proto-oncogene BCL3	Peroxisome proliferator-activated receptor (PPAR)γ; interferon regulatory factor 4 (IRF4)	[30–32],[33]
miR-21	Almost all carcinomas and hematolgogical malignancies	Plays important roles in the oncogenic process by targeting PTEN, PDCD4, and BTG2	TGF-β; STAT3; IFN	[6, 34–37]
miR-196	Pancreatic adenocarcinoma, breast cancer, ovarian cancer, oral cancer, Crohn’s disease	Promoting cell migration and invasion; promoting proliferation of and suppressing apoptosis	IFN-β; IκBα	[38] [39–41]
miR-210	Breast cancer, squamous cell carcinoma, renal cancer, sarcoma, bladder cancer, glioblastoma	Plays important roles in the regulation of cell growth, angiogenesis, and apoptosis	Hypoxia inducing factors (HIFs); IL-6; TNF	[42–44]
miR-126	Inflammatory bowel disease (IBD); its expression is suppressed in cancers of the gastrointestinal tract, genital tract, breast, thyroid, and lung	Plays a key role in autoimmune and inflammatory diseases; can inhibit the progression of some cancers via negative control of proliferation, migration, invasion, and cell survival	NF-κB; vascular endothelial growth factor A (VEGF-A)	[45–47]
miR-132	Upregulated during the inflammatory phase of wound repair; glioma	Critical regulator of skin wound healing; inhibits Smad7 expression	TGF-β1; TGF-β2	[48, 49]
miR-146	Pancreatic carcinoma, papillary thyroid carcinoma, gastric cancer, breast cancer, non-small cell lung cancer	Control of the inflammatory response of cells of the innate immune system; plays a role in the development and maintenance of neoplastic processes	TNF; IL-1	[50, 51]

Aberrant miRNA expression leads to developmental abnormalities and diseases; however, the processes regulating miRNA biogenesis are largely unknown. miRNAs are transcribed as long and capped polyadenylated pri-miRNAs. The pri-miRNA is cropped into a hairpin-shaped pre-miRNA by the Drosha complex. Next, the pre-miRNA is translocated to the nucleus by exportin-5 and further processed by the Dicer complex. The resulting miRNA is dissociated and incorporated into the RNA-induced silencing complex (RISC), where it functions in gene silencing by promoting the degradation of target mRNAs or by translational inhibition. The identification of mechanisms of miRNA biogenesis regulation revealed that various factors or growth factor signaling pathways control every step of the miRNA biogenesis pathway [52].

miR-155, which was the first miRNA shown to play an oncogenic role [53], is overexpressed in a variety of tumors such as leukemia/lymphoma, breast, colon, lung, pancreatic, and gastric tumors [25, 26]. Enhanced expression of miR-155 is often associated with increased cytokine expression, a prominent feature of inflammatory processes [27, 54, 55]. For example, lipopolysaccharide (LPS)/TNF-α stimulation results in the upregulation of miR-155 and downregulation of miR-125b [27]. miR-21 is upregulated in almost all carcinomas and hematological malignancies [6] and is induced in macrophages and blood mononuclear cells upon lipopolysaccharide (LPS) challenge [34] and in mammary epithelial cells by inflammatory signals [35]. miR-210 links inflammatory signals with the hypoxic microenvironment, as it is induced in response to low oxygen and inhibited by the cytokines IL-6 and TNF [42]. On the other hand, inflammatory cytokines can be modulated by miRNAs. Several miRNAs, such as miR-126, miR-132, miR-146, miR-155, and miR-221, are important transcriptional regulators of certain inflammation-related mediators [8].

## Upregulated miRNAs by the TGF-β/Smad signaling pathway

**Fig. 2:**
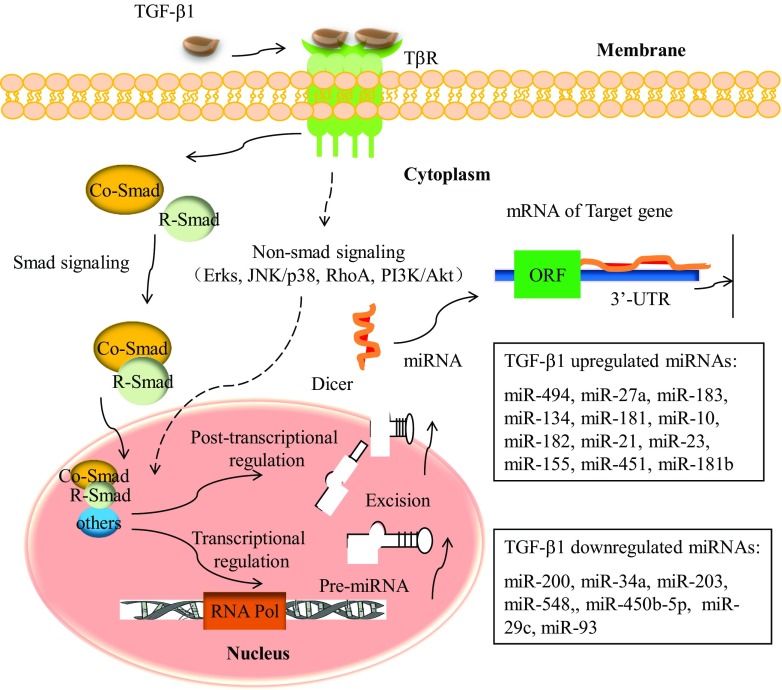
The TGF-β signaling pathway and related miRNAs. Ligand binding to the TGF-β receptor (TβR)-mediated heterotetramer formation. This signal is relayed through the nucleus via restricted Smad (R-Smad). Activated R-Smads complex with common Smads (Co-Smad, such as Smad-4) translocate to the nucleus and alter miRNA transcription. In addition, TGF-β signaling alteration of miRNA transcription may depend on non-Smad molecules, such as ERKs, JNK/p38, RhoA, and PI3K/Akt. The mechanisms of TGF-β signaling-mediated miRNA expression are either transcriptional or post-transcriptional

TGF-β function is dependent on tissue type and the epigenetic background of cells [56]. One prominent feature of TGF-β biology is its dual role: It functions as a tumor suppressor in the early stages of tumorigenesis, whereas it promotes tumor cell metastasis in advanced stages [56, 57]. The interaction between TGF-β signaling and miRNAs has been investigated extensively, and studies suggest that the TGF-β pathway can either inhibit or enhance miRNA maturation [58–61]. Figure 2 shows a brief outline of the miRNAs associated with the TGF-β/Smad signaling pathway in cancer.

Davis et al. [62] was the first to describe mechanisms of miRNA expression modulation and showed that TGF-β treatment resulted in the upregulation of pre-miRNAs and mature miRNAs, but not that of pri-miRNAs. Smad proteins have been shown to control the transcription of miRNA-coding genes by binding to miRNA promoter genes [59]. Smads control miRNA biogenesis by two different mechanisms that involve complex Smad2–3 binding to Smad4 or not. The Smad2–3 complex is translocated to the nucleus, where it is recruited by the Drosha/DGCR8 microprocessor complex and promotes miRNA maturation [23, 58, 59, 63, 64]. However, the mechanism underlying the translocation of the Smad2–3 complex to the nucleus remains undetermined. The most prominent miRNAs upregulated by TGF-β signaling are miR-21, miR-181, miR-494, and miR-10.

### miR-21

A meta-analysis revealed that high miR-21 levels are related to poor overall survival, suggesting that miR-21 is a prognostic factor for poor survival in cancer patients [65]. TGF-β-mediated induction of miR-21 was firstly found to be independent of Smad4, although it is dependent on the R-Smads [62]. TGF-β-induced miR-21 levels are markedly higher in transformed cells, and miR-21 contributes to chemoresistance in breast cancer cells by targeting the MutS homolog 2 [66]. In bladder tumors, in accordance to the observed similarity between TGF-β variants and miR-21 gene expression alterations, treatment of the bladder cancer cell line 5637 with recombinant TGF-β induced a significant upregulation of miR-21 [67] and confirmed the correlation between TGF-β and miR-21 expression in cancer. Furthermore, miR-21 is regarded as a potential therapeutic target [68]. Wang et al. [69] investigated the effect of ursolic acid (UA) on human gliomas and found that UA inhibits cell growth by inducing apoptosis in U251 cells via a UA-triggered TGF-β1/miR-21/PDCD4 pathway.

### miR-181

Wang et al. [70] demonstrated that TGF-β-mediated upregulation of hepatic miR-181b promotes hepatocarcinogenesis by targeting the tissue inhibitor of metalloprotease 3 (TIMP3) [70]. In this article, the authors showed that the upregulation of hepatic TGF-β and its downstream Smad mediators in liver nuclear extracts correlated with elevated miR-181b/d in mice fed a choline-deficient and amino acid-defined (CDAA) diet. The levels of miR-181b increased upon exposure of hepatic cells to TGF-β and were inhibited by siRNA-mediated depletion of Smad4, demonstrating the role of TGF-β signaling in miR-181b biogenesis. Moreover, miR-181a had a direct effect on inducing hepatocyte epithelial-mesenchymal transition (EMT) and was able to replace TGF-β-induced effects in vitro [71]. In the field of breast cancer research, Wang et al. [72] observed that exposure to TGF-β increased the population of breast cancer cells that can form mammospheres in suspension, and this was mediated by miR-181, which was upregulated by TGF-β at the post-transcriptional level. Neel et al. [73] identified miR-181 as a potent regulator of TGF-β signaling in human breast cancer and found that miR-181 is the Smad2/3-dependent downstream target of TGF-β signaling. Furthermore, their data demonstrates that miR-181 is required for TGF-β-mediated cell migration and invasion, as silencing miR-181 expression significantly antagonizes the pro-invasive effects of this growth factor.

### miR-494

Myeloid-derived suppressor cells (MDSCs) potently suppress the anti-tumor immune responses that favor tumor angiogenesis and metastasis; however, the molecular networks regulating the accumulation of tumor-expanded MDSCs are largely unknown. Liu et al. [74] revealed that TGF-β-upregulated miR-494 is required for the accumulation and activity of MDSCs via targeting of phosphatase and tensin homolog (PTEN). In pancreatic ductal adenocarcinomas (PDACs), dysregulation of β-catenin and the transcriptional activator FOXM1 mediates oncogenesis, although the underlying mechanism remained unclear until Li et al.[75] showed that loss of Smad4 in PDAC cells leads to reduced levels of miR-494, increased levels of FOXM1, and nuclear localization of β-catenin. Therefore, miR-494 might be developed as a prognostic marker for patients with PDAC or as a therapeutic target.

### miR-10b

Ma et al. [76] firstly reported miR-10b upregulated in breast cancer and showed that the level of miR-10b expression in primary breast carcinomas correlates with clinical progression. Then, the results of follow-up studies supported these results [77, 78]. Recently, miR-10b is identified as a target gene of TGF-β1, which upregulated miR-10b, promotes EMT, and increases the metastatic potential of breast cancer cells [79]. Ouyang et al. [80] demonstrated that overexpression of miR-10b accelerated pancreatic cancer cell (PCC) proliferation and tumor growth in an orthotopic model. In this article, the authors showed that miR-10b and TGF-β function together to markedly increase cell invasion, and this effect is blocked by the combination of erlotinib and SB505124, a type I TGF-β receptor inhibitor. miR-10b also enhances the stimulatory effects of TGF-β on cell migration and EMT. Therefore, therapeutic targeting of miR-10b in malignancy may block growth-promoting and antagonize the metastatic process at various levels.

### Other microRNAs: miR-27a/miR-183/miR-182/miR-155/miR-451

TGF-β-associated miR-27a inhibits dendritic cell-mediated differentiation of Th1 and Th17 cells by modulating TAB3, p38 mitogen-activated protein kinase (MAPK), MAP2K4, and MAP2K7 [81]. TGF-β-inducible miR-183 silences tumor-associated natural killer cells [82]. TGF-β-induced miR-182 induces an aggressive phenotype by targeting CYLD in glioma subsets [83]. TGF-β upregulates miR-155 through Smad4 and contributes to epithelial cell plasticity by targeting RhoA in breast cancer [84]. Moreover, Smad upregulates miR-451 and drives glioblastoma stem cell to no-stem cell transformation, leading to reduced glioblastoma tumorigenicity [85].

## Downregulated miRNAs by the TGF-β/Smad signaling pathway

With respect to cancer, miRNAs are often located in fragile genomic sites and are therefore typically downregulated in tumors [6]; inhibition of miRNA biogenesis tends to enhance tumorigenesis [86], raising the possibility that miRNA re-expression potentially represents an effective therapy for cancer [87]. The examples below are representative miRNAs downregulated by TGF-β signaling.

### miR-200

Gregory et al. [88] firstly reported that all five members of the miR-200 family (miR-200a, miR-200b, miR-200c, miR-141, and miR-429) are markedly downregulated in cells that undergo EMT in response to TGF-β. These authors showed that these miRNAs cooperatively regulate the expression of the E-cadherin transcriptional repressors zinc finger E-box binding homeobox 1 (ZEB1; also known as deltaEF1) and SIP1 (also known as ZEB2), factors previously implicated in EMT and tumor metastasis. Truong et al. [89] later found that TGF-β-downregulated miR-200 elicits migratory behavior by increasing ZEB2 in triple-negative breast cancer. Recently, a TGF-β-miR200-Mig6 pathway was shown to induce resistance to EGFR inhibitors in lung and pancreatic cancers [90].

### miR-34a

Altered miR-34 expression has been determined to be involved in the pathogenesis of many cancers [91]. The miR-34a is one member of miR-34 family, which of miR-34a, miR-34b, and miR-34c. miR-34a is encoded by its own transcript, whereas miR-34b and miR-34c share a common primary transcript. Ectopic re-expression of miR-34a in both primary and tumor-derived cell lines is correlated with cycle arrest, apoptosis, and cell growth inhibition [92]. Yang et al. [93] demonstrated that elevated TGF-β activity associated with the persistent presence of hepatitis B virus in liver tissues suppresses the expression of miR-34a, leading to enhanced production of the chemokine CCL22, which recruits regulatory T cells to facilitate immune escape. This indicates that restoring the tumor suppressor miR-34, as well as blocking TGF-β signaling, may provide a novel molecular therapy for the treatment of cancer.

### miR-203

miR-203 has been identified as a skin-specific keratinocyte-derived miRNA that is located at chromosome 14q32-33 [94]. miR-203 expression is significantly downregulated in laryngeal squamous cell carcinoma and is correlated with poor differentiation, advanced clinical stage, and lymph node metastasis [95]. Xu et al. [96] revealed that miR-203 is downregulated in renal cancer and confirmed that FGF2 is a direct target of miR-203. Ding et al. [97] demonstrated that TGF-β represses the expression of miR-203 to promote EMT and tumor metastasis. In this paper, miR-203 was significantly downregulated in highly metastatic breast cancer cells, and restoration of miR-203 in highly metastatic breast cancer cells inhibited tumor cell invasion in vitro and lung metastatic colonization in vivo by repressing SNAI2.

### miR-584

miR-584 was shown to decrease cell motility through the inhibition of Rho-associated coiled-coil-containing protein kinase 1 (ROCK-1) in renal cell carcinoma (RCC) cell lines, and the expression of miR-584 was inversely correlated with that of ROCK-1 in RCC tissues [98]. These results suggest that miR-584 functions as a new tumor suppressor miRNA in RCC by downregulating ROCK-1. Fils-Aime et al. [99] identified miR-584, a potential tumor suppressor, as a novel target of TGF-β and found that miR-584 expression is negatively regulated by this growth factor in a number of breast cancer cells. These authors found that inhibition of miR-584 expression by TGF-β is required for cell migration, as overexpression of ectopic miR-584 reversed TGF-β-induced cell migration. They further identified protein phosphatase and actin regulator 1 (PHACTR1) as a downstream target of miR-584 and found that TGF-β-mediated inhibition of miR-584 leads to increased expression of PHACTR1.

### miR-450b-5p

TGF-β1 was shown to exert its function by suppressing miR-450b-5p, which significantly inhibited the growth of rhabdomyosarcoma (RMS) and promoted the expression of MyoD. Using a bioinformatics approach, we identified ecto-NOX disulfide-thiol exchanger 2 (ENOX2) and paired box 9 (PAX9) as targets of miR-450b-5p. These results suggest that disrupting the TGF-β1 suppression of miR-450b-5p or knockdown of ENOX2 and PAX9 are effective approaches in inducing RMS MyoD. Furthermore, we found that the Smad3 and Smad4 pathways, but not Smad2, are the principal mediators of TGF-β1 suppression of miR-450b-5p [100].

## miRNAs regulate the TGF-β signaling pathway

Most members of the TGF-β pathway are known to be targeted by one or more miRNAs [23]. After the identification of TGF-β1, TGF-βR (I and II) and Smads were found to be dysregulated in most cancers, whereas miRNAs potentially targeting these molecules are downregulated. The impact of miRNA on canonical Smad signaling has been investigated extensively.

### TGF-β1

Interactions between miRNAs and TGF-β1 signaling have been validated experimentally with suggesting that miRNAs influence the TGF-β1 signaling at multiple levels. A few such interactions have been demonstrated. For example, Martin et al. [101] identified multiple binding sites for miR-744 located in the proximal TGF-β1 3′-UTR. miR-744 transfection inhibited endogenous TGF-β1, which given the pleiotropic nature of cellular responses to TGF-β1 is potentially significant. Dogar et al. [102] showed that reduced levels of the oncomirs miR-18a and miR-24 accounted for the observed derepression of two TGF-β1 processing factors, thrombospondin-1 (THBS1) and furin, respectively, which suggested a novel mechanism by which latent TGF-β1, thrombospondin 1, and furin form a miRNA-mediated regulatory feedback loop. Ectopic expression of latent TGF-β1 reduces THBS1 protein expression and is associated with increased expression of let-7 and miR-18a in cells [103]. These data suggest an inverse correlation between THBS1 and latent TGF-β1 expression levels possibly involving miRNAs.

### TGF-βR I and II

Downregulation of miR-30 or miR-200 upregulates TGF-βR I and Smad2 to direct the EMT and invasive potential of anaplastic thyroid carcinomas [104]. In breast cancer cells, miR-128a promotes letrozole resistance by targeting TGF-βR I [105]. To date, many miRNAs targeting TGF-βR II have been identified in different malignancies and shown to contribute to tumor progression. miR-590-5p promotes the proliferation and invasion of human hepatocellular carcinoma cells [106], and miR-106b induces the migration and invasion of colorectal cancer cells [107]. High expression of miR-370 in gastric carcinoma tissues is associated with increased nodal metastasis and advanced clinical stage compared with controls [108], and miR-211 promotes the tumorigenesis of head and neck carcinomas [109]. Moreover, miR-21 induces stemness by targeting TGF-βR II in colon cancer cells [110] and promotes tumor development by targeting of TGF-βR II and Smad2/3 in prostate cancer [111]. The miR-520/373 family functions as a tumor suppressor in estrogen receptor negative breast cancer by targeting NF-κB and TGF-βR II [112]. Downregulation of miR-655 increases ZEB1 and TGF-βR II levels to accelerate cancer progression [113]. Recently, the miR-17 family was shown to reverse cisplatin resistance and suppress metastasis by targeting TGF-βR II in NSCLC [114].

### Smads

miR-148a attenuates the cancer stem cell-like properties of hepatocellular carcinoma cells by targeting Smad2 [115]. miR-99a and miR-99b enhance cell migration and adhesion of normal murine mammary gland cells by targeting Smad3 [116]. miR-92b functions as a potential oncogene by targeting Smad3 and promotes glioblastoma cell proliferation [117].

miR-146b-5p increases cell proliferation and cell cycle arrest by repressing Smad4 in thyroid cancer [118]. miR-199a acts as a negative regulator of TGF-β signaling by targeting Smad4 and plays an oncogenic role in gastric cancer [119]. miR-130a-mediated downregulation of Smad4 reduces the sensitivity to TGF-β1 stimulation in granulocytic precursors [120]. Moreover, miR-130a/301a/454 functions as oncogenes by targeting Smad4 to enhance cell proliferation and migration in human colorectal cancer [121]. As a tumor suppressor miRNA, miR-34a acts as a tumor suppressor by targeting Smad4 in proneural subtype glioblastoma [122]. Downregulated miR-146a increases Smad4 and affects cell proliferation in response to retinoid acid induction in an acute promyelocytic leukemia cell line [123].

miR-155 renders diffuse large B cell lymphoma (DLBCL) cells resistant to the growth inhibitory effects of TGF-β1 and BMP by targeting Smad5 [124]. Moreover, miR-155 controls RB phosphorylation in normal and malignant B lymphocytes via the non-canonical TGF-β1/Smad5 signaling module [125]. Kaposi’s sarcoma-associated herpesvirus-encoded miR-K12-11 facilitates cell proliferation through the suppression of Smad5 [126]. The miR-106b-25 cluster targets the inhibitory Smad7 protein, resulting in the overexpression of TGF-βR I, and activates TGF-β signaling to induce EMT and the tumor-promoting effect of Six1 treatment in human breast cancer cells [127]. Moreover, miR-216a/217 induces EMT by targeting PTEN and Smad7 to promote drug resistance and recurrence of liver cancer [128].

### Other mechanisms

The miR-106b-25/miR-17-92 cluster interferes with cell cycle arrest and apoptosis to resist TGF-β tumor suppression [129]. Moreover, the miR-106b-25 cluster inhibits the TGF-β tumor suppressor pathway, interfering with the expression of CDKN1A and BCL2L11 (Bim) in gastric cancer [130]. The miR-17-92 miRNA cluster was shown to act both upstream and downstream of psmad2, resulting in the downregulation of multiple key effectors of the TGF-β signaling pathway and in the inhibition of TGF-β-responsive genes in neuroblastoma cells [131]. miR-183 inhibits TGF-β1-induced apoptosis by downregulating PDCD4 in human hepatocellular carcinoma cells [132]. Moreover, miR-204 and miR-379 target IL-11 and downregulate the expression of several genes involved in TGF-β signaling, including PTGS2 in bone metastatic breast cancer cells [133]. miR-127-3p inhibits cancer cell proliferation and activates TGF-β signaling by targeting SKI in glioblastoma [134].

## miRNAs and non-Smad pathways in malignancy

In addition to the canonical Smad pathway, the effects of TGF-β are largely dependent on the interactions between numerous non-canonical signaling factors such as ERK, p38, RhoA, and phosphoinositide 3 kinase (PI3K) [135, 136]. The effect of the canonical signaling pathway on miRNA expression regulation has been investigated and was reviewed by Blahna et al. [58]. However, miRNA alterations of non-canonical signaling in malignancies have been characterized to a lesser extent. Since differences in miRNA expression levels will impact non-canonical signaling, these different miRNAs could exert anti-tumor effects by altering the TGF-β signaling pathway, highlighting miRNAs targeting non-canonical signaling as potential therapeutic targets. For example, miR-27a, as an inhibitor of MAPK as well as JNK during dendritic cell-mediated accumulation of Tregs in vivo, accelerated tumor growth by inducing the accumulation of immune cells, indicating that miR-27a is a potential target for cancer therapy [81].

## Exosomic miRNAs in the tumor microenvironment

Alongside with well-known pathways by which cells can communicate, considerable attention is now being given to the role of extracellular vesicles (EVs) which have been shown to contain nucleic acids in form of miRNAs [137, 138]. More importantly, the discovery that EVs can deliver miRNAs to target cells, raising the possibility that such exosomes could work as a novel category of intercellular communicators [137, 139, 140]. Some researchers demonstrated that exosome-associated miRNAs are involved in the metastatic potential of malignancy. For example, EVs derived from metastatic breast cancer cells were shown to be able to deliver miR-200 to non-metastatic cells [141]. As outlined in a recent review [142], the tumor cells can “educate” the surrounding environment from normal to pro-inflammatory and pro-tumorigenic through exosome-dependent signals other than well-known cross talk.

## Future directions and concluding remarks

Inflammatory circuits can differ considerably between different tumors in terms of cellular networks and cytokines. Understanding the diversity of the inflammatory microenvironment is instrumental to the design of therapeutic approaches targeting this microenvironment. Many anti-inflammatory drugs inhibit the activity of cytokines and tumor development in preclinical and clinical settings associated with cancer [143–145]. These conventional therapies mainly depend on genetic alterations of cancer cells and target cancer-associated inflammatory cell receptors or cytokines and their receptors [146].

Inflammatory cytokines, including TGF-β, play important roles in the regulation of tumor progression. Owing to the pleiotropic effects of TGF-β on the tumor microenvironment, targeting TGF-β signaling to directly treat cancer progression remains controversial. As described in this review, the TGF-β signaling pathway plays a critical role in the regulation of miRNA biogenesis. Given that a single miRNA can modulate the expression of hundreds of target genes, the regulation of miRNAs by the TGF-β signaling pathway could be critical for the modulation of the tumor microenvironment.

Several studies have investigated the role of TGF-β-related miRNAs in cancer and revealed the different mechanisms underlying the interaction between TGF-β and miRNAs in cancer. In TGF-β overexpressing cancer tissues, upregulated miRNAs may play a tumor-promoting role by downregulating the expression of tumor-suppressor genes. Therefore, downregulating the expression of these miRNAs and/or upregulating the expression of target genes could be a strategy for the treatment of cancer. On the other hand, the downregulation or inhibition of certain miRNAs in cancer may lead to oncogene overexpression and tumor-promoting effects. In this case, increasing the expression of these miRNAs and/or downregulating the expression of target genes could be applied for the treatment of cancer. In brief, understanding the molecular and epigenetic mechanisms underlying the relationship between inflammatory cytokine signaling and miRNAs in cancer may facilitate the development of new therapeutic strategies targeting the tumor microenvironment.
